# Neo-Dermis Formation and Graft Timing After ADM Reconstruction: A Cohort Study with Histological Validation

**DOI:** 10.3390/jfb16120469

**Published:** 2025-12-18

**Authors:** Daniel Pit, Teodora Hoinoiu, Bogdan Hoinoiu, Cristian Suciu, Panche Taskov, Zorin Petrisor Crainiceanu, Daciana Grujic, Isabela Caizer-Gaitan, Miruna Samfireag, Oana Suciu, Razvan Bardan

**Affiliations:** 1 Doctoral School, “Victor Babes” University of Medicine and Pharmacy, E. Murgu Square, No. 2, 300041 Timisoara, Romania; daniel.pit@umft.ro (D.P.); panche.taskov@umft.ro (P.T.); 2 Discipline of Clinical Skills, Department I Nursing, “Victor Babes” University of Medicine and Pharmacy, E. Murgu Square, No. 2, 300041 Timisoara, Romania; isabela.caizer@umft.ro (I.C.-G.); samfireag.miruna@umft.ro (M.S.); 3 Center for Advanced Research in Cardiovascular Pathology and Hemostaseology, “Victor Babes” University of Medicine and Pharmacy, E. Murgu Square, No. 2, 300041 Timisoara, Romania; 4 Burn Unit, Plastic and Reconstructive Surgery Department, Casa Austria, “Pius Brinzeu” Emergency County Clinical Hospital, 300723 Timisoara, Romania; crainiceanu.zorin@umft.ro (Z.P.C.); grujic.daciana@umft.ro (D.G.); 5 Department of Oral Rehabilitation and Dental Emergencies, Faculty of Dentistry, “Victor Babes” University of Medicine and Pharmacy, E. Murgu Square, No. 2, 300041 Timisoara, Romania; 6 Interdisciplinary Research Center for Dental Medical Research, Lasers and Innovative Technologies, Revolutiei 1989 Avenue No. 9, 300070 Timisoara, Romania; 7 Department of Microscopic Morphology/Histology, “Victor Babes” University of Medicine and Pharmacy, E. Murgu Square, No. 2, 300041 Timisoara, Romania; cristian_suciu@umft.ro; 8 Angiogenesis Research Centre, “Victor Babes” University of Medicine and Pharmacy, 300041 Timisoara, Romania; 9 Pathology Department, “Pius Brinzeu” Emergency County Clinical Hospital Timisoara, 300723 Timisoara, Romania; 10 Plastic Surgery Department, “Victor Babes” University of Medicine and Pharmacy, E. Murgu Square, No. 2, 300041 Timisoara, Romania; 11 Department of Rehabilitation, Physical Medicine and Rheumatology, “Victor Babes” University of Medicine and Pharmacy, E. Murgu Square, No. 2, 300041 Timisoara, Romania; oanasuciu78@umft.ro; 12 Department of Urology, “Victor Babes” University of Medicine and Pharmacy, E. Murgu Square, No. 2, 300041 Timisoara, Romania; razvan.bardan@umft.ro

**Keywords:** acellular dermal matrix, Integra, Nevelia, dermal regeneration template, split-thickness skin graft, graft timing, neovascularization, CD105 (endoglin), D2-40 (podoplanin), burns, oncologic reconstruction, length of stay

## Abstract

Acellular dermal matrices (ADMs) are widely used in soft-tissue reconstruction, yet the optimal timing for split-thickness skin grafting (STSG) remains unsettled. We conducted a single-center retrospective cohort study (January 2023–August 2025) of adults undergoing ADM-based reconstruction with Integra^®^ Double Layer (IDL), Integra^®^ Single Layer (ISL), or Nevelia^®^. Primary endpoints included length of stay (LOS), STSG requirement and timing, and in-hospital complications; secondary endpoints included spontaneous epithelialization. Prespecified adjusted analyses (linear/logistic models) controlled for age, sex, etiology, anatomical site, diabetes/PAOD, smoking, wound size (when available), wound contamination, and matrix type. Histology and immunohistochemistry (H&E, Masson trichrome, CD105, D2-40) assessed matrix integration and vascular/lymphatic maturation. Seventy-five patients were included (IDL n = 40; ISL n = 20; Nevelia n = 15). On multivariable analysis, matrix type was not an independent predictor of LOS (ISL vs. IDL β = +2.84 days, 95% CI −17.34 to +23.02; Nevelia vs. IDL β = −4.49 days, 95% CI −16.24 to +7.26). Complications were infrequent (6/75, 8.0%) and comparable across matrices; spontaneous epithelialization occurred in 3/75 patients (4.0%). A day-14 grafting strategy, applied only after documented clinical integration, was feasible in 30/75 (40.0%) patients without excess complications. Histology/IHC at 3–4 weeks demonstrated CD105-positive, perfused capillary networks with abundant collagen; at 4–6 weeks, D2-40-positive lymphatic structures confirmed progressive neo-dermis maturation, supporting the biological plausibility of earlier grafting once integration criteria are met. In this cohort, outcomes were broadly similar across matrices after adjustment. A criteria-based early STSG approach (~day 14) appears safe and operationally advantageous when integration is confirmed, while a minority of defects may heal without grafting. Prospective multicenter studies with standardized scar/functional measures and cost analyses are needed to refine patient selection and graft timing strategies.

## 1. Introduction

Complex soft-tissue defects arising after oncologic excision, trauma, burns, or ischemic complications remain difficult to reconstruct when local tissue is insufficient or when immediate flap coverage is not feasible. In such cases, acellular dermal matrices (ADMs) offer a reliable dermal substitute that supports neovascularization and cellular infiltration, enabling staged resurfacing with split-thickness skin grafts (STSGs) or, in select cases, secondary epithelialization [1]. Among the clinically used devices, the Integra^®^ Dermal Regeneration Template is one of the best-established products, while other dermal substitutes, including the Nevelia^®^ Dermal Substitute, differ in architecture and temporary epidermal coverage, which may influence handling characteristics, integration dynamics, and timing of definitive coverage [2,3,4]. Although fewer studies evaluate Nevelia^®^ compared with Integra^®^, available data indicate rapid integration and adequate vascularization, particularly in post-oncologic excision defects and certain chronic wounds [5,6,7].

Despite their broad use across reconstructive indications, several gaps in evidence remain. First, there is no consensus on the optimal interval between ADM placement and STSG, with current practice ranging between two and three weeks based largely on bedside assessment rather than standardized criteria. Second, the comparative performance of commonly used matrices across heterogeneous etiologies and anatomical sites is insufficiently characterized, and most reports lack adjustment for potential confounders such as indication, comorbidities, and wound location. Third, outcomes are often reported qualitatively (e.g., “satisfactory,” “excellent”) without standardized metrics or robust effect estimates for clinically relevant endpoints such as length of stay (LOS), complications, or STSG timing and necessity. Finally, although histology and immunohistochemistry (IHC) are frequently used to evaluate integration, the relationship between microscopic markers of angiogenesis/lymphangiogenesis and practical clinical decision points—such as readiness for grafting—remains poorly systematized.

The primary aim of this study was to compare clinical outcomes and the optimal timing of split-thickness skin grafting among three clinically used ADMs (Integra^®^ Double Layer, Integra^®^ Single Layer, and Nevelia^®^), using prespecified adjusted analyses to account for relevant clinical variables. A secondary objective was to perform histological and immunohistochemical evaluations of ADM integration and vascular maturation to provide biological context supporting the feasibility of earlier grafting.

## 2. Materials and Methods

### 2.1. Materials

Three acellular dermal matrices (ADMs) were used in this study: Integra^®^ Double Layer, Integra^®^ Single Layer, and Nevelia^®^.

The Integra^®^ Dermal Regeneration Template (IDRT; Integra LifeSciences, Princeton, NJ, USA) is a bilayer acellular construct initially developed for full-thickness burn management and subsequently applied to traumatic defects, oncologic resections, chronic wounds, and complex reconstructive scenarios. The dermal component consists of a porous scaffold made of cross-linked type I bovine collagen combined with shark chondroitin-6-sulfate, designed to support cellular infiltration, neovascularization, and organized neodermal formation. A semi-permeable silicone membrane provides temporary epidermal protection. Crosslinking enhances mechanical stability and resistance to premature enzymatic degradation [8,9,10].

The Integra^®^ Dermal Regeneration Template—Single Layer is a three-dimensional porous scaffold composed of cross-linked bovine tendon collagen and chondroitin-6-sulfate. Engineered with controlled porosity and biodegradation kinetics, it provides biomechanical stability while supporting fibroblast infiltration, neovascularization, and progressive replacement by host-derived neodermal tissue. Available in thin and regular thicknesses, it may be used alone or combined with the bilayer template to optimize dermal thickness in wounds of varying depth [11].

NEVELIA^®^ (Symatese, Chaponost, France) is a bilayer dermal regeneration matrix composed of purified, stabilized bovine type I collagen arranged in a highly fibrous three-dimensional porous architecture optimized for cellular adhesion, fibroblast migration, and neodermal development. A controlled crosslinking profile and a pore size of approximately 100 μm promote balanced resorption and vascularized neo-dermis formation within 2–3 weeks. The collagen layer is covered by a polyester-reinforced silicone membrane that provides temporary epidermal protection. Nevelia^®^ is indicated for burns, trauma, chronic wounds, and oncologic defects and is also used in pediatric reconstruction [12].

### 2.2. Study Design and Objectives

This study was approved by the Ethics Committee of the “Pius Brînzeu” County Emergency Clinical Hospital, Timișoara, Romania (resolution no. 566/03 September 2025). This study adhered to the ethical guidelines set forth in the Helsinki Declaration for medical research involving human subjects, as revised at the 64th WMA General Assembly in Fortaleza, Brazil, in October 2013.

We performed a single-center retrospective cohort study at the “Casa Austria” Plastic, Reconstructive Surgery, and Burns Clinic (January 2023–August 2025). Adult patients undergoing staged reconstruction with Integra^®^ Double Layer (IDL), Integra^®^ Single Layer (ISL), or Nevelia^®^ were included.

The study objectives were to (i) quantify clinical integration time, STSG requirement/timing, complications, and length of stay (LOS); (ii) compare outcomes between matrices using adjusted analyses for etiology, anatomical site, and comorbidities; (iii) provide histological and immunohistochemical correlates of ADM integration.

We hypothesized that (1) early grafting (~day 14) after documented integration is not inferior to later grafting (≥21 days) regarding complications and may shorten LOS; (2) matrix-related differences in unadjusted outcomes would diminish after adjustment for case-mix; (3) a subset of defects would achieve secondary epithelialization without STSG, with frequency dependent on indication and anatomical site.

### 2.3. Eligibility and Participants

The inclusion criteria were adult patients (≥18 years old) with a soft-tissue defect requiring staged reconstruction with ADM due to post-traumatic, post-tumor excision, post-burn, ischemic/diabetic/peripheral arterial occlusive disease (PAOD) etiologies, or other complex defects.

The exclusion criteria were active, uncontrolled infection at the defect site, severe comorbidities contraindicating surgery, refusal to participate, and inability to ensure postoperative clinical follow-up.

A total of 75 patients met the criteria: 15 had post-traumatic defects, 30 had post-tumor excision, 24 had post-burn, five were ischemic-related, and one had stage IV Dupuytren’s release. The ADM type was customized for each patient: IDL for 40 patients, ISL for 20 patients, and Nevelia^®^ for 15 patients.

### 2.4. Operating Protocol

All defects underwent standardized wound bed preparation (sharp excision/debridement of non-viable tissue, hemostasis, contouring to healthy margins, and negative-pressure therapy when indicated). The selected ADM (IDL/ISL/Nevelia^®^) was tailored, secured with non-absorbable sutures or staples, and covered with a compressive sterile dressing per the device instructions and unit protocol. Prophylactic antibiotics and analgesia were administered according to institutional standards. Dressings remained undisturbed until postoperative day 7, after which they were changed according to local evolution. Patients were monitored every 24–48 h. A biopsy of the integrating ADM was performed on day 14 to assess matrix incorporation. In oncologic cases, this biopsy did not evaluate margins, which were already confirmed at the time of excision.

#### Clinical Definition of ADM “Integration” and Decision Rule for Grafting

Clinical integration was defined a priori as meeting all of the following criteria: (i) uniform adherence of the matrix to the wound bed without dead space or shear; (ii) capillary bleeding on gentle curettage or pin-prick testing through a small access point in the silicone layer; (iii) absence of infection indicators (no purulent exudate, malodor, or progressive erythema).

If all criteria were satisfied at the ~14-day assessment, patients proceeded to STSG; if criteria were incompletely met, grafting was deferred, with continued local care, and typically performed at ≥21 days, once criteria were fulfilled. In our cohort, 30 patients underwent STSG at approximately 2 weeks and the remainder at approximately 3 weeks according to this rule.

### 2.5. Histopathological and Immunohistochemical Examination

Primary tissue fragments were processed in the Pathological Anatomy Department of Timisoara Clinical Emergency County Hospital according to standard protocols. The specimens were fixed in 10% neutral buffered formalin for 24 h and processed using an automated tissue processor. For morphological and immunohistochemical assessments, 5 µm sections were cut from paraffin blocks. Morphological evaluation was performed on sections stained with hematoxylin–eosin (H&E) using the Leica automated system.

#### Immunohistochemical Staining

Immunohistochemistry was performed on the Leica Bond platform. Antigen retrieval used Epitope Retrieval Solution 2 for 20 min (Leica Biosystems, Newcastle Ltd., Newcastle Upon Tyne, UK) followed by the Bond Polymer Refine Detection System, which comprises peroxide block, post-primary reagent, polymer, DAB chromogen, and a hematoxylin counterstain. After a 5 min peroxide block, primary antibodies were applied for 20 min each as follows: CD34 (Leica Bond RTU, mouse anti-human, clone QBEnd/10), CD31 (Leica Bond RTU, mouse anti-human, clone JC70A), CD68 (Leica Bond RTU, mouse anti-human, clone 514H12), CD105/Endoglin (Santa Cruz Biotechnology, Heidelberg, Germany), mouse anti-human, clone A-8, 1:100 dilution), and podoplanin D2-40 (Dako Flex RTU, mouse anti-human, clone D2-40). Post-primary and polymer incubations were each 8 min, DAB (mixed refine) was 10 min, and hematoxylin counterstaining was 5 min.

Special histochemical stains included H&E to assess overall architecture and matrix integration, Masson’s trichrome to highlight collagen fibers, and orcein to evaluate elastic fibers and dermal matrix remodeling. The immunohistochemical panel was selected to contextualize tissue-level changes during integration: CD105 as a marker of active angiogenesis; CD31 and CD34 as endothelial markers for microvascular density and distribution; and D2-40 (podoplanin) as a marker of lymphatic endothelium and lymphangiogenesis.

Quality control included appropriate on-slide positive controls and negative controls (isotype or primary-antibody omission) for each run, and staining intensity and distribution were reviewed by a pathologist blinded to the clinical time point. Representative micrographs were acquired with uniform magnification, and scale bars are provided in the figure panels.

### 2.6. Variables, Covariates, and Statistical Analysis

From the medical record we extracted age, sex, etiology (trauma, oncologic excision, burn, ischemic/diabetic/PAOD, other), anatomical site (scalp; head/neck; trunk; upper limb; lower limb; foot), diabetes and/or PAOD, smoking status, wound size (cm^2^) when documented, wound contamination at the index procedure, and matrix type (IDL/ISL/Nevelia^®^). These prespecified variables served as covariates in adjusted analyses. Data entry was performed using Microsoft Excel (version 16.103.3.), and statistical analysis was performed using IBM SPSS Statistics v31.

Continuous variables were checked for distribution with Shapiro–Wilk and visual inspection and are reported as median [IQR] or mean ± SD as appropriate; categorical variables as n (%). Between-matrix comparisons used ANOVA (with Welch’s correction if needed) or Kruskal–Wallis for continuous data and χ^2^ or Fisher’s exact tests for categorical data; significant omnibus tests were followed by Holm–Bonferroni-adjusted pairwise contrasts.

To address confounding by indication and site, we fitted logistic regression for STSG requirement (yes/no) and any complication (yes/no), linear regression for length of stay (LOS, days) with HC3 robust standard errors when residuals suggested heteroskedasticity, and Kaplan–Meier/log-rank with Cox proportional hazards for time to integration when exact dates were available. All models included the prespecified covariates above. We assessed model assumptions and diagnostics (linearity of the logit for continuous predictors, multicollinearity by VIF, residual patterns for linear models, and proportional hazards by Schoenfeld residuals). Effect sizes are presented as odds ratios (ORs), β-coefficients, or hazard ratios (HRs) with 95% confidence intervals and two-sided *p*-values; α = 0.05. Missing covariate data < 10% were handled by complete-case analysis; when ≥10%, we planned multiple imputation (m = 20) under a missing-at-random assumption with pooling by Rubin’s rules. This convenience cohort had no a priori sample size calculation.

## 3. Results

We included 75 adults undergoing staged reconstruction with acellular dermal matrices: IDL in 40 (53.3%), ISL in 20 (26.7%), and Nevelia^®^ in 15 (20.0%) cases. Baseline demographics were broadly comparable across groups (Table 1). Median age (years) was 63.0 [46.5–73.2] for IDL, 65.0 [51.5–71.2] for ISL, and 61.0 [54.0–72.5] for Nevelia^®^, with female proportions of 50.0%, 45.0%, and 26.7%, respectively. Case-mix by etiology (Table 2) showed that ISL was preferentially used in burns (75.0%), whereas IDL and Nevelia^®^ were more often applied after oncologic excision or trauma. Anatomical site distribution is summarized in Table 3; upper and lower limb defects predominated overall, with a non-negligible fraction categorized as “other/NA” where documentation was less specific.

Hospital length of stay (LOS) varied across matrices in unadjusted comparisons: IDL 3.0 days [1.0–12.0], ISL 18.0 days [11.5–30.5], and Nevelia^®^ 3.0 days [2.0–16.5] (Kruskal–Wallis *p* = 0.0038; Table 4). Given the enrichment of burn indications and distinct site profiles in the ISL group, we fitted a prespecified adjusted linear model. After controlling for etiology, anatomical site, age, and sex, matrix type was not independently associated with LOS (ISL vs. IDL: β = +2.84 days, 95% CI −17.34 to +23.02, *p* = 0.783; Nevelia^®^ vs. IDL: β = −4.49 days, 95% CI −16.24 to +7.26, *p* = 0.454; Table 4). Thus, the longer LOS observed with ISL appears largely attributable to case-mix rather than a device effect.

All patients were reassessed on day 14 using predefined integration criteria. Thirty patients (40.0%) underwent STSG on day 14, while forty-five (60.0%) underwent STSG at ≥21 days after additional local care. A small subset healed without grafting (3/75, 4.0%), with no significant difference across matrices (χ^2^ *p* = 0.545; Table 4). In-hospital complications during the index admission were uncommon (6/75; 8.0%) and did not differ significantly by matrix (IDL 7.5%, ISL 0%, Nevelia^®^ 20.0%; χ^2^ *p* = 0.096; Table 4). Recorded events included infection, matrix breakdown/lysis, and reoperation, managed with local care, systemic antibiotics, and, when indicated, surgical revision.

Taken together, these data indicate that although LOS differs across matrices in crude analyses—partly driven by indication and site—matrix choice itself was not an independent predictor of LOS once key covariates were accounted for. Rates of secondary epithelialization without STSG were low and similar between devices, and complication rates were small and not significantly different across matrices.

### 3.1. Pathology Findings

**Figure 1:**
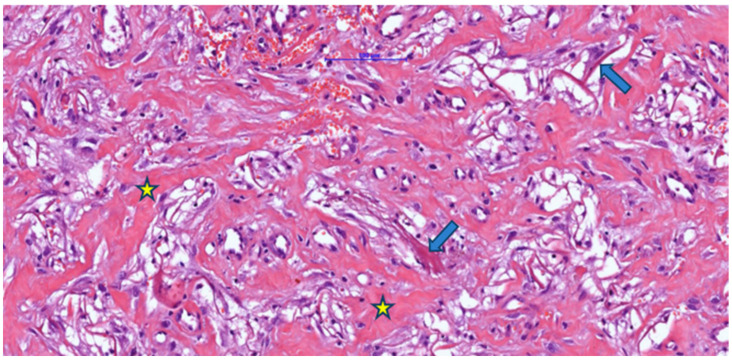
Post-implantation dermal tissue fragments with Integra in the neovascularization phase (weeks 3–4), showing remnants (arrow) of eosinophilic material (Integra) with the formation of numerous collagen fibers (asterisk) associated with a discrete mononuclear inflammatory infiltrate and vascular lumens, some of which contain blood (H&E).

Morphological evaluation of skin fragments with Integra implants showed clear signs of the neovascularization phase and early remodeling at 3–4 weeks after implantation (Figure 1). These included eosinophilic, sinuous matrix remnants that maintained polygonal or angular contours, indicating residual porosity of the original scaffold. In addition, type I collagen fibers with an eosinophilic appearance on H&E were present, arranged in bundles in some areas and intertwined with newly formed capillaries with clear lumens containing red blood cells, confirming vascular infiltration at this stage.

**Figure 2:**
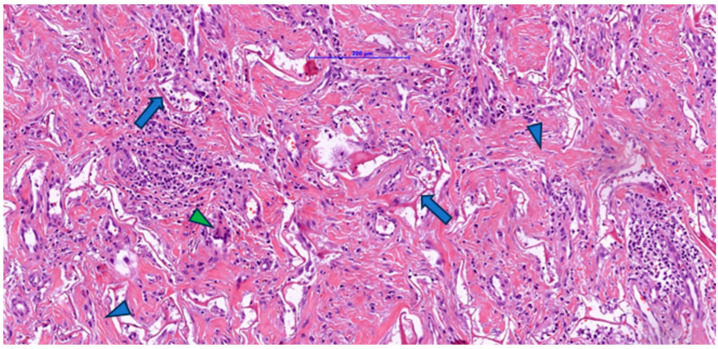
Post-implantation dermal tissue fragments with Integra in the neovascularization phase showed remnants (arrow) of eosinophilic material (Integra), numerous collagen fibers (blue arrowhead), and a prominent lymphocytic, plasmacytic, and macrophage inflammatory infiltrate, including multinucleated giant cells (green arrowhead).

Associated with these structures, dispersed fibroblastic cells with a fusiform appearance and elongated nuclei were arranged parallel to the collagen fibers, indicating active cellular colonization and endogenous collagen deposition. The examined fragment showed pale/clear lacunae—empty spaces corresponding to initial pores or areas where the matrix had been partially degraded—some lined by cells with mesenchymal features, suggesting ongoing integration. Figure 1 illustrates minimal inflammation with rare perivascular mononuclear cells, whereas Figure 2 shows foci of inflammatory infiltrate composed of lymphocytes, macrophages, and plasma cells, as well as multinucleated foreign-body-type giant cells.

**Figure 3:**
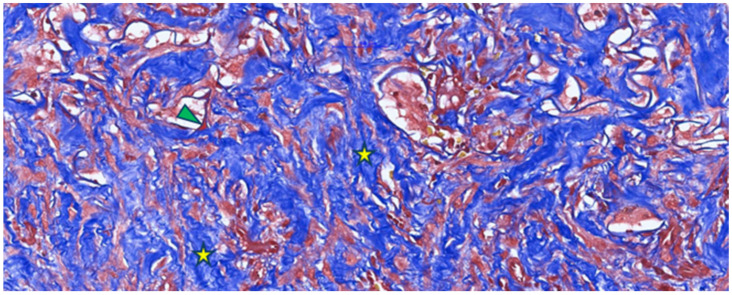
Masson’s trichrome staining of a post-implantation Integra-type skin fragment in the neovascularization and collagen-formation phase, highlighting numerous collagen fibers (type I collagen) in blue (yellow asterisk) with a disordered arrangement and limited areas of Integra-type material (arrowhead).

Masson’s trichrome staining (Figure 3) revealed numerous blue-stained collagen fibers, either individually or in bundles, reflecting collagen production by fibroblasts colonizing the Integra matrix. An interconnected arrangement of collagen fibers was observed in a disorganized pattern resembling normal dermis, rather than the parallel arrangement typical of hypertrophic scars. Small remnants of dermal matrix with a reddish-brown appearance were interspersed among the blue collagen fibers.

**Figure 4:**
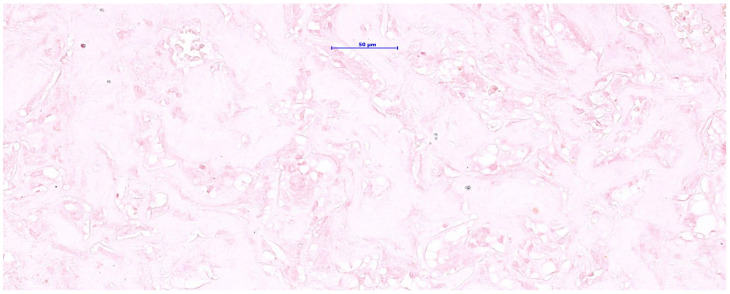
Orcein staining for elastic fibers showed a negative reaction (absence of elastic fibers) in a skin graft with integrin during the neovascularization phase.

Elastic fibers were absent on orcein staining in fragments with Integra implants during the neovascularization phase (Figure 4).

**Figure 5:**
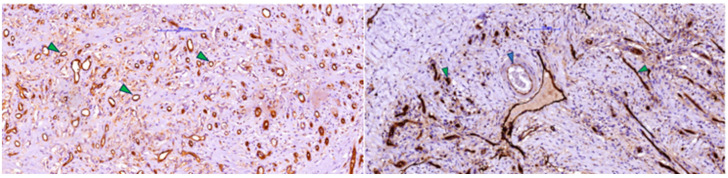
(**Left**) Immunoreaction for CD105 (endoglin) was positive in activated/dividing endothelial cells in post-implant dermal scar tissue with Integra, showing numerous small-caliber capillary-like vessels (arrowhead), consistent with neoangiogenesis and immature vessels. (**Right**) Scar tissue fragment (from deep tissue) showing several small neoformed vessels with intensely positive endothelium (green arrowhead) for CD-105 and a small artery with muscular wall (blue arrowhead) with weak positive reaction for endoglin (CD-105).

Building on these morphological aspects, immunostaining for CD105/Endoglin (Figure 5) provided functional evidence of the vascularization observed on H&E. CD105 highlighted proliferative endothelium in newly formed vessels, revealing a dense capillary network with approximately 70 vascular lumens distributed throughout the matrix. Vascular profiles ranged from round or oval lumens measuring 8–12 µm to branched structures with thin walls lined by 1–2 strongly positive endothelial cells, consistent with immature yet viable endothelium. Many lumens contained blood, supporting true microvascular perfusion rather than merely the presence of vascular channels.

The rich vascularization observed with CD105 immunostaining is consistent with the abundance of newly formed collagen highlighted in blue on Masson’s trichrome (Figure 3), as fibroblasts receive sufficient oxygen and nutrients for active matrix synthesis. Overall, these findings indicate that by 3–4 weeks post-Integra implantation, the matrix has transitioned from an inert scaffold to a regenerated, richly vascularized dermis colonized by fibroblasts and in an advanced phase of collagen remodeling—conditions favorable for subsequent epithelial grafting.

**Figure 6:**
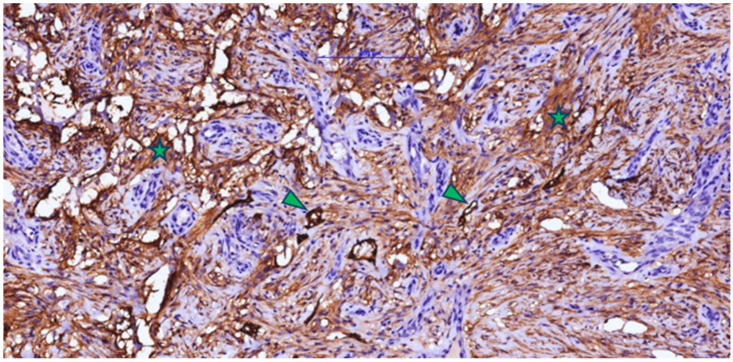
Post-implant scar tissue with Integra in the neovascularization and connective fiber formation phases, showing a strong positive reaction for D2-40 in fibroblastic/myofibroblastic cells (asterisk) and lymphatic endothelium (arrowhead) (lymphangiogenesis).

The presence of D2-40 (podoplanin)-positive vascular structures (Figure 6) without intraluminal blood supports the early formation of a functional lymphatic plexus in addition to the intense blood vascularization (CD105 positive). Although less numerous than CD105-positive vessels, these lymphatic channels facilitate drainage of excess fluid and cytokines, reduce edema, and promote the decline of inflammatory infiltrate, improving antigen transport to loco-regional lymph nodes and suggesting that the regenerated dermis is acquiring the structure and function of normal connective tissue. D2-40 immunolabeling also revealed a population of spindle-shaped cells positive for podoplanin—activated fibroblasts/myofibroblasts that transiently express podoplanin during repair—whose presence, together with collagen bundles, is consistent with an active remodeling phase and a potential contribution to controlled wound contraction.

An essential aspect of ADM reconstruction is the selection of optimal graft timing. In most patients, around day 14 post-application, the dermal matrix was clinically integrated and the silicone layer could be removed easily, revealing a healthy, well-vascularized neoderm suitable for grafting. Manufacturers generally recommend grafting at 21–28 days after application, depending on local evolution. In our clinical practice, however, grafting at three weeks or later was sometimes associated with minor local issues (serous or serosanguinous secretions beneath the silicone sheet, perilesional erythema, and occasionally increased fragility of the integrated tissue). These observations support the hypothesis that in cases with favorable evolution, earlier grafting (around day 14) can be performed safely, potentially avoiding minor complications related to bacterial colonization or hypergranulation.

### 3.2. Case Presentations

**Figure 7:**
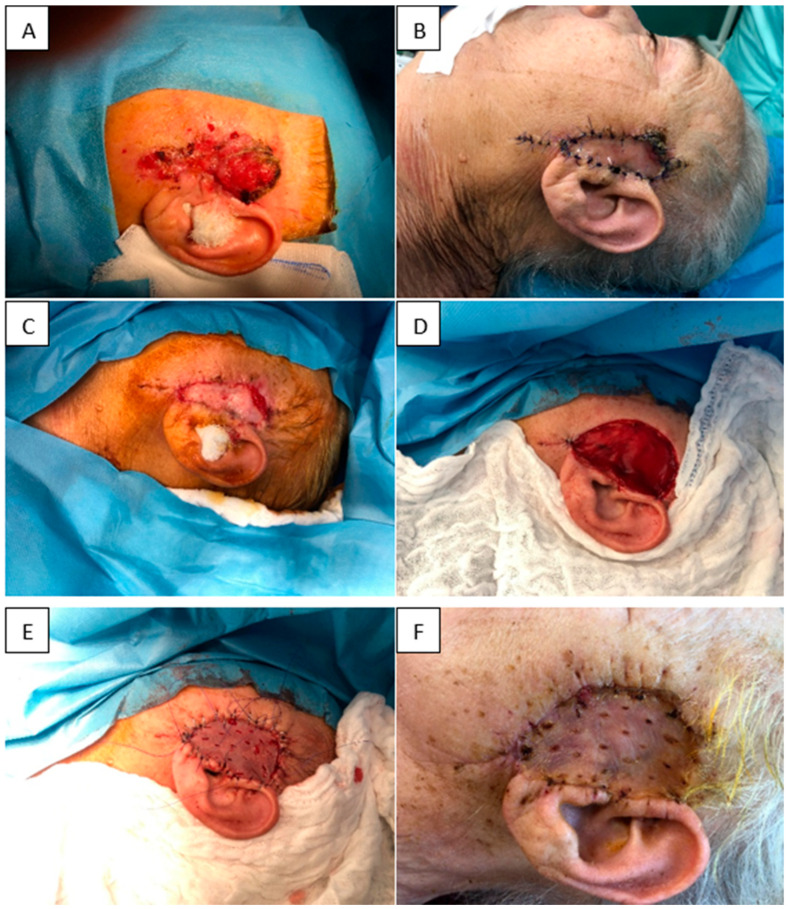
(**A**) Initial tumor. (**B**) Nevelia^®^ dermal matrix at 1 week. (**C**) Nevelia^®^ integrated at 3 weeks. (**D**) Re-excision with removal of the matrix and wider margins. (**E**) STSG application. (**F**) STSG integrated at 3 weeks postoperatively.

**Figure 8:**
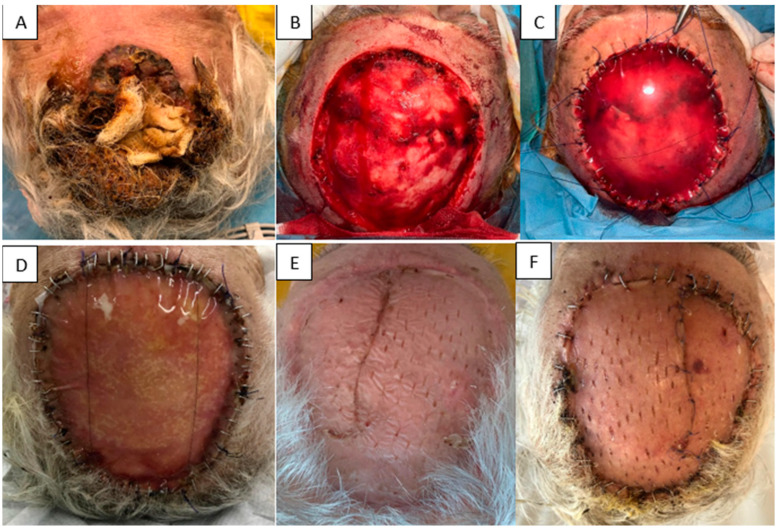
(**A**) Initial tumor. (**B**) Resulting defect. (**C**) Application of Integra^®^ Double Layer. (**D**) Two weeks after IDL application. (**E**) STSG at 6 months. (**F**) STSG at 1 week post-grafting.

**Figure 9:**
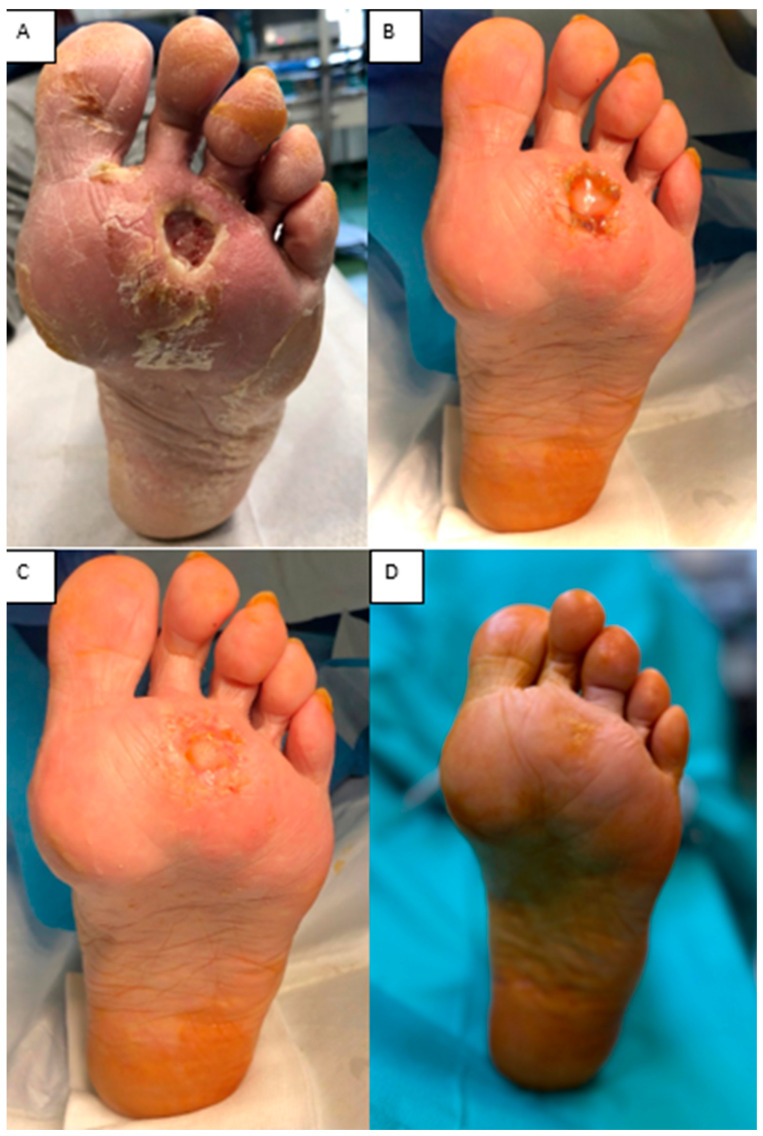
(**A**) Plantar ulcer and soft-tissue defect. (**B**) Integra^®^ at 3 weeks, with and without the silicone layer. (**C**) Spontaneous epithelialization of the matrix. (**D**) Clinical aspect at 1 year.

**Figure 10:**
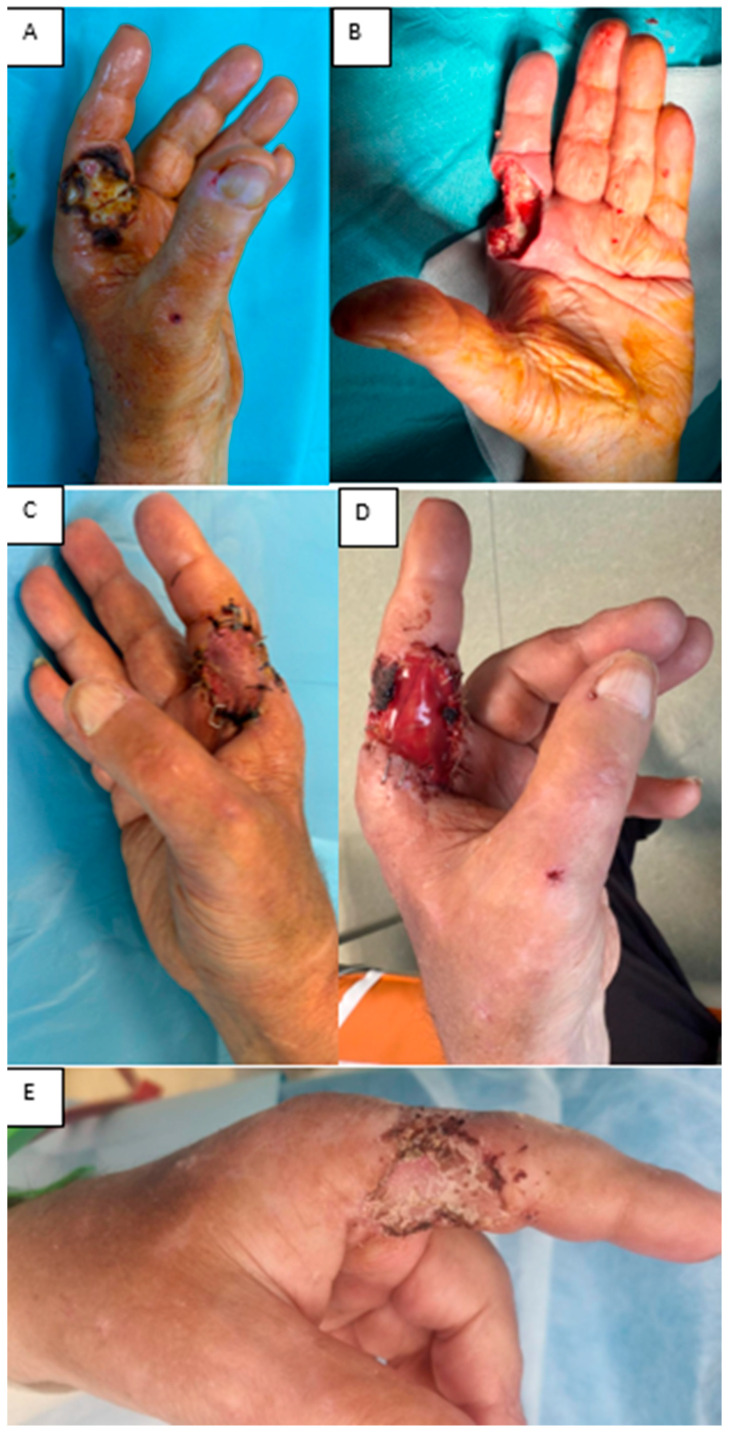
(**A**) Dry finger necrosis. (**B**) Post-debridement defect. (**C**) Integra^®^ DL at 1 week. (**D**) STSG at 1 week post-grafting. (**E**) STSG at 1 month.

(i) A 73-year-old woman with multiple comorbidities presented with an ulcerated cutaneous tumor in the left preauricular region. The lesion was excised and the resulting defect immediately covered with Nevelia^®^ dermal matrix. Histopathology revealed positive resection margins, indicating residual tumor. Three weeks later, re-excision with wider oncologic margins and removal of the initial matrix was performed, and the new defect was reconstructed with Integra^®^ Single Layer plus split-thickness skin grafting in the same session. At one week postoperatively, the skin graft showed complete integration (100%) with no local complications. This case illustrates the feasibility of sequential dermal matrix application combined with timely grafting in complex facial oncologic defects in elderly patients (Figure 7).

(ii) An 86-year-old woman with significant cardiovascular disease presented with a large, ulcerated, superinfected cutaneous tumor in the frontoparietal region. The tumor was excised with oncologic margins and the defect immediately reconstructed with Integra^®^ Double Layer. At 2 weeks, the matrix appeared fully integrated, adherent, and secretion-free. A split-thickness skin graft harvested from the thigh was applied over the matrix. One week later, the graft showed 100% integration and firm adherence. At 6 months, the graft remained soft and elastic, without contracture. This case demonstrates successful staged reconstruction with IDL and delayed grafting, achieving favorable functional and aesthetic outcomes (Figure 8).

(iii) A 70-year-old woman with diabetes mellitus, peripheral arterial disease, and neuropathy presented with a plantar ulcer and soft-tissue defect. After thorough debridement, the defect was covered with Integra^®^ Dermal Layer (IDL). At 3 weeks, the matrix was fully integrated, and the defect healed by secondary intention without the need for skin grafting. This case shows that, in carefully selected plantar defects with significant comorbidities, dermal matrix application alone can achieve complete wound closure (Figure 9).

(iv) A 61-year-old man with end-stage chronic kidney disease on long-term dialysis, with a functioning arteriovenous fistula in the affected limb, presented with dry necrosis of the radial aspect of the index finger (F1–F2) and tendon/neurovascular exposure after debridement. An Integra^®^ Double Layer matrix was applied to promote vascularized tissue over the exposed structures. Two weeks later, a full-thickness skin graft from the iliac fossa was used to close the defect. At one week, the graft showed 98% integration, no secretions, and complete coverage of vital structures, preserving finger function. This case underscores the value of staged IDL plus full-thickness grafting for complex digital defects in high-risk patients (Figure 10).

**Figure 11:**
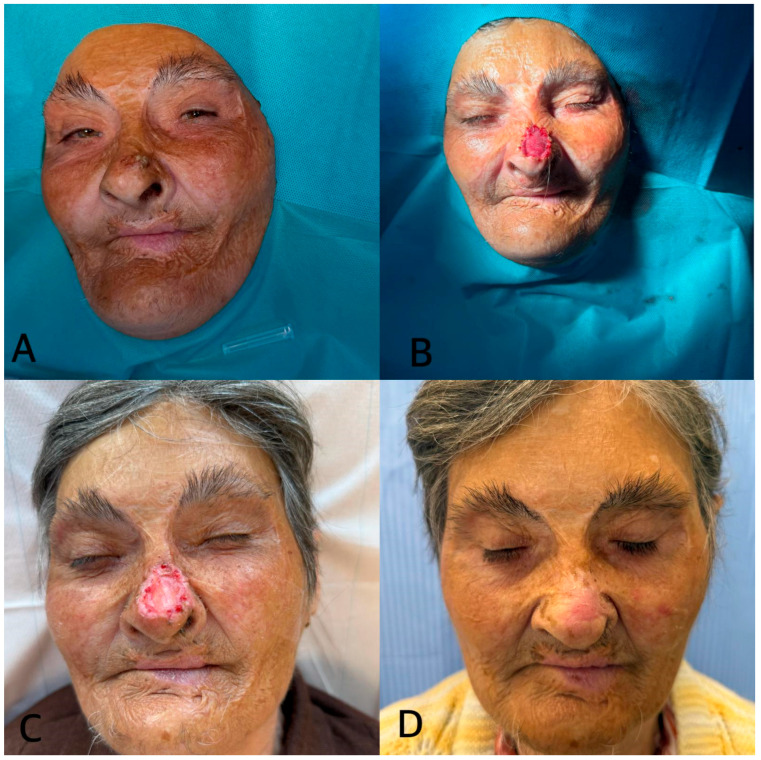
(**A**) Initial nasal lesion. (**B**) Application of Integra^®^ DL. (**C**) Two weeks post-op. (**D**) Spontaneous epithelialization at 1 month.

**Figure 12:**
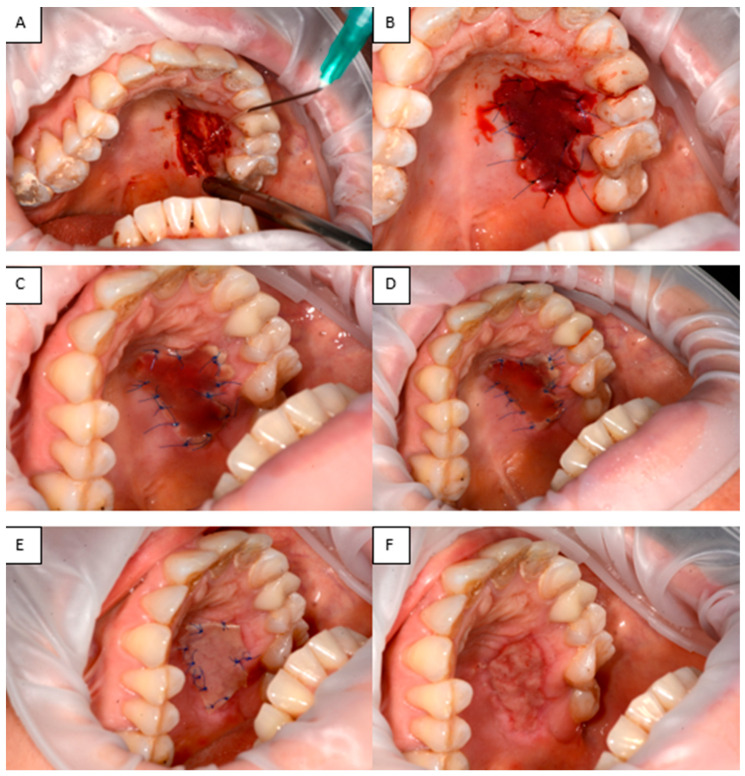
(**A**) Defect in the hard palate. (**B**) Integra^®^ applied to the palatal defect. (**C**–**E**) Appearance of Integra^®^ at 3, 5, and 7 days, respectively. (**F**) Healed tissue.

(v) A 72-year-old woman with severe remote facial burns presented with a suspected basal cell carcinoma (BCC) of the nose. The lesion was excised with oncologic margins and the defect immediately covered with Integra^®^ Double Layer. Grafting was postponed until histopathological confirmation of clear margins. One week after matrix application, Integra^®^ appeared well integrated, adherent, and secretion-free. At one month, the nasal defect had completely healed by secondary intention, without grafting. This case illustrates that Integra^®^ DL can support spontaneous healing of nasal oncologic defects, avoiding additional graft procedures and achieving good functional and aesthetic results even in previously burned tissue (Figure 11).

(vi) A 48-year-old woman with a history of a dental implant-supported crown in the lateral right mandibular region (tooth #28, 29, 30) loaded 6 months prior, presented with a deficiency of keratinized tissue and vestibular depth. Consequently, we opted to perform free gingival graft harvested from the palatal aspect of the left molar area. This procedure aims to address the primary consequences of buccal flap advancement during dental implant insertion and subsequent bone augmentation, which often results in the loss of keratinized mucosa and vestibular depth. Such loss can lead to a mismatch in mucosal color and texture around the dental implant compared to those around adjacent natural teeth. The recipient site was prepared, and the free graft was harvested from the palate. The graft, approximately 2 mm thick, was tailored to meet the specific needs of the recipient area. A sterile paper template was created to match the dimensions of the FGG. The graft donor area extended from the canine to the second molar, with a horizontal cut made approximately 1.5–2 mm from the tooth margin. After harvesting the graft, bleeding was controlled by applying pressure with sterile gauze soaked in saline. Following graft application to the recipient site, the palatal wound was measured using a periodontal probe (mm^2^) and covered with a shaped Integra^®^ Double Layer artificial dermal matrix. Unlike traditional dressing methods, which may shift or be removed, the artificial dermal matrix was securely fastened with sutures, minimizing micromovements and mechanical trauma at the donor sites. This mechanical protection likely facilitated uninterrupted epithelial migration and safeguarded the early clot from distortion (Figure 12).

## 4. Discussion

Acellular dermal matrices (ADMs) have become indispensable tools for soft-tissue reconstruction in plastic, oncologic, traumatic, and reconstructive surgery. In our cohort of 75 patients, unadjusted comparisons suggested longer hospital stay in ISL cases; however, in prespecified adjusted analyses (controlling for etiology, anatomical site, age, and sex), matrix type was not independently associated with LOS (ISL vs. IDL β = +2.84 days, 95% CI −17.34 to +23.02; Nevelia^®^ vs. IDL β = −4.49 days, 95% CI −16.24 to +7.26). Secondary epithelialization without STSG was rare (3/75; 4.0%), and in-hospital complications were infrequent (6/75; 8.0%), with no statistically significant differences between matrices. Clinically, a day-14 grafting strategy, applied only after documented integration, was feasible in a substantial proportion of cases and was not associated with higher complication rates.

ADMs are processed biological scaffolds devoid of viable cells but preserving the native collagen and dermal architecture, thereby promoting effective host integration. Our findings emphasize the importance of timing for skin-graft application: grafting around day 14 post-ADM placement, once predefined integration criteria were fulfilled, appeared safe and was associated with shorter ward time in everyday practice. In contrast, delaying grafting to ≥21 days was sometimes associated with minor local issues (e.g., serous or serosanguinous secretions beneath the silicone layer, perilesional erythema) without a clear reduction in complication rates.

This observation is consistent with evidence indicating that neovascularization of the applied matrix typically occurs between 7 and 14 days post-application (Chang et al., 2019 [13]), providing the physiological substrate for vascular ingrowth and cellular migration. Our biopsy data support this temporal window and demonstrate its operational relevance in clinical decision-making. Furthermore, a clinical trial evaluating Integra^®^ for post-burn defect coverage has reported safe grafting within the 10–14-day interval, reinforcing this period as a practical decision point for selected patients. Based on these convergent data, our group aimed to introduce a standardized, criteria-based protocol that enables reliable grafting approximately 14 days after ADM placement in appropriately evolving wounds [14,15,16,17].

Early ADM integration triggers a cascade of biological processes, including angiogenesis, fibroblast proliferation, extracellular matrix deposition, and re-epithelialization. Thus, ADMs act not only as passive scaffolds but as bioactive microenvironments that recruit host cells, support keratinocyte migration, and facilitate formation of a functional, interconnected capillary network. Histological analysis in our series corroborates this concept: at 3–4 weeks, we observed dense CD105-positive capillary networks with perfused lumens and abundant collagen on Masson’s trichrome, and at 4–6 weeks, D2-40-positive lymphatic structures. These findings are in line with prior reports showing robust cellular infiltration and endothelial marker expression (e.g., CD31) in ADMs during the early integration phase. The capacity of ADMs to serve as “living templates” has been well described, particularly for MatriDerm, Integra^®^, and AlloDerm [13,14,15,16].

From an economic and logistical perspective, achieving effective ADM integration within two weeks may translate into reduced overall LOS. In our practice, patients undergoing STSG around day 14 generally had shorter hospitalizations than those grafted later, consistent with reports suggesting that early grafting after ADM placement can reduce hospitalization and reintervention rates [18,19]. Although formal cost-effectiveness analyses were beyond the scope of this study, such efficiencies are likely to improve patient experience and reduce the risk of nosocomial complications.

Another key advantage of ADMs is the potential reduction in surgical risk. By obviating or reducing the need for dermal/fascial donor sites, ADMs can lower donor-site morbidity and shorten operative time, which may in turn decrease postoperative pain—particularly relevant in frail or multimorbid patients. Several studies have associated ADM use with lower rates of seroma, marginal skin necrosis, and postoperative scar contracture, especially in complex facial and cervical reconstructions where both mobility and cosmesis are critical [20].

While our study and others highlight the versatility of ADMs, device-specific trade-offs have been reported. Paganelli et al. and others describe MatriDerm as more prone to scar retraction, whereas Integra^®^ has been associated with slightly higher rates of infectious complications, delayed resorption, or granulomatous foreign-body reactions. Nonetheless, histology often shows broadly similar neo-dermal composition between products. Consequently, both remain valid options in modern dermato-oncologic surgery, and device selection is best individualized according to defect size and site, patient comorbidities (e.g., diabetes, peripheral vascular disease), and exposure of critical structures [21,22]. In our cohort, this principle guided matrix choice: Integra^®^ Double Layer was commonly selected for larger defects requiring structural support, while Nevelia^®^ tended to be used for smaller, well-vascularized wounds in which faster integration was anticipated.

Beyond direct wound-closure outcomes, early ADM integration may accelerate functional recovery. In defects near joints or regions exposed to mechanical stress, early rehabilitation is crucial for preventing stiffness and contractures. By providing elastic, conformable coverage capable of tolerating external forces, ADMs can facilitate early mobilization and reduce the risk of wound dehiscence. In extensive oral mucosal defects following oncologic resection, biosynthetic dermal matrices offer a promising alternative to microvascular free tissue transfer, with reduced donor-site morbidity and support for mucosal regeneration. Existing literature suggests that Integra^®^ is generally well tolerated, non-allergenic, and capable of supporting complete re-epithelialization via its collagen–glycosaminoglycan porous architecture and semi-permeable silicone membrane [1,23,24].

Importantly, our histology and IHC data directly support the “2–4 weeks neo-dermis” concept in this series. By 3–4 weeks post-Integra^®^ implantation, we documented a richly vascularized dermis (CD105-positive proliferative endothelium with perfused lumens), abundant collagen deposition (Masson’s trichrome), and, by 4–6 weeks, D2-40-positive lymphatic structures. These findings provide a mechanistic rationale for the observed safety and practicality of earlier (~day-14) grafting once clinical integration criteria are met, suggesting that the vascular and lymphatic thresholds required for graft take are achieved within this timeframe [13,25,26,27].

Integra^®^ Dermal Matrix has also been used in various surgical fields, including mucogingival procedures, to address tissue defects where minimizing donor-site morbidity and scarring is particularly important [28,29]. Its use in intraoral applications, such as palatal defects, has been reported as a viable alternative to traditional grafts, allowing effective re-epithelialization with reduced discomfort in oral mucosal defects [30]. In mucogingival surgery, acellular dermal matrices, including Integra^®^, may be employed to increase keratinized tissue and cover recession defects. The integration process has been shown to lead to increased bands of keratinized mucosa and controlled gingival inflammation over time [28]. Additionally, ADM use in complex reconstructions such as palatal fistula repairs has been associated with reduced recurrence rates, further supporting its value in challenging anatomical locations [31].

Despite these clear advantages, the broader adoption of ADMs remains constrained by cost and availability, the need for experienced multidisciplinary teams, and the requirement for strict adherence to application protocols (excluding cases with active infection or severe ischemia), as well as meticulous postoperative monitoring and wound care [18,19].

### Limitations

This retrospective, single-center study is subject to several limitations. First, its observational design makes it vulnerable to residual confounding despite prespecified adjusted analyses. Second, we did not collect standardized scar or functional outcome scales, which precludes objective evaluation of aesthetic and functional endpoints and limits comparability with studies using validated instruments. Third, surgeon preference for matrix choice may have introduced selection bias (e.g., ISL preferentially selected for burns, IDL for larger structurally demanding defects), which we attempted to mitigate through adjusted models but cannot fully exclude.

Nevertheless, these real-world data provide useful preliminary insights into the performance of different dermal matrices and into the feasibility of a criteria-based early grafting strategy. Future research should focus on prospective, multicenter designs with standardized aesthetic and functional outcome measures, formal cost-effectiveness analyses, and predefined matrix allocation protocols, as well as direct head-to-head comparisons of ADM products to validate and refine these findings.

## 5. Conclusions

Acellular dermal matrices offer a safe and effective approach for reconstructing complex soft tissue defects, resulting in improved outcomes and reduced donor-site morbidity. When feasible, early split-thickness skin grafting (around day 14) is recommended to optimize integration and minimize complications. However, selected patients may experience spontaneous epithelialization, highlighting the inherent regenerative potential of ADMs. Future multicenter, prospective studies are essential to establish standardized treatment protocols, directly compare the various ADM types, and rigorously assess their long-term functional and economic outcomes, which will facilitate optimized ADM selection in soft tissue reconstruction.

This study makes several contributions to existing literature. It offers an adjusted comparative evaluation of three commonly used acellular dermal matrices, overcoming the limitations of previous non-comparative reports. It also assessed the feasibility of an earlier, criteria-based STSG strategy in a real-world cohort. Moreover, the inclusion of histology and immunohistochemistry (CD105, D2-40) provides biological insight into the timeline of vascular and lymphatic maturation, supporting the plausibility of earlier grafting once the integration criteria are met. Finally, we intend to further expand this work through ongoing clinical and histopathological investigations to validate and refine these findings in larger prospective cohorts.

## Figures and Tables

**Table 1:** Baseline demographics by matrix.

Matrix	N	Age, Years (Median [IQR])	Female, %
IDL	40	63.0 [46.5–73.2]	50.0%
ISL	20	65.0 [51.5–71.2]	45.0%
Nevelia	15	61.0 [54.0–72.5]	26.7%

Continuous variables are presented as median [IQR] and categorical variables as %.

**Table 2:** Etiology by matrix (% within matrix; n).

Matrix	Burn	Oncologic Excision	Trauma	Ischemic /Diabetic Foot
IDL (n = 40)	17.5% (7)	60.0% (24)	17.5% (7)	5.0% (2)
ISL (n = 20)	75.0% (15)	5.0% (1)	15.0% (3)	5.0% (1)
Nevelia (n = 15)	20.0% (3)	33.3% (5)	33.3% (5)	13.3% (2)

**Table 3:** Anatomical site by matrix (% within matrix).

Matrix	Scalp	Head/Neck	Trunk	Upper Limb	Lower Limb	Foot	Other/ NA
IDL (n = 40)	10.0	2.5	7.5	22.5	12.5	7.5	37.5
ISL (n = 20)	5.0	0.0	5.0	5.0	5.0	10.0	70.0
Nevelia (n = 15)	0.0	6.7	0.0	20.0	6.7	6.7	60.0

Within-matrix percentages. The full granular distribution at specific locations is provided in Table S1 (Supplementary Information).

**Table 4:** Outcomes by matrix.

Outcome	IDL (n = 40)	ISL (n = 20)	Nevelia (n = 15)	Overall (N = 75)	*p*-Value *
Length of stay, days (median [IQR])	3.0 [1.0–12.0]	18.0 [11.5–30.5]	3.0 [2.0–16.5]	8.0 [2.0–21.0]	0.0038
Healed without STSG, n (%)	2 (5.0%)	0 (0.0%)	1 (6.7%)	3 (4.0%)	0.5446
Any complication, n (%)	3 (7.5%)	0 (0.0%)	3 (20.0%)	6 (8.0%)	0.0960

* Kruskal–Wallis test for length of stay; χ^2^ test for categorical outcomes (Fisher’s exact test when expected count < 5).

## Data Availability

The original contributions of this study are included in this article. Further inquiries can be directed to the corresponding authors.

## Supplementary Materials

The following supporting information can be downloaded at: https://www.mdpi.com/article/10.3390/jfb16120469/s1. Table S1 sumarisation of Etiology defect, anatomical site of defect, second stage of autografting timing differences between ADM, graft tickness and histological examination. Table S2 Our results in regard of the literature findings. References [32,33,34,35,36,37,38] are cited in the supplementary materials.
