# Supplementary material for: Neo-Dermis Formation and Graft Timing After ADM Reconstruction: A Cohort Study with Histological Validation
Source: J Funct Biomater. 2025 Dec 18;16(12):469. doi: 10.3390/jfb16120469 (PMC12733933; doi:10.3390/jfb16120469)
Supplement: Supplementary file 1 [file jfb-16-00469-s001.zip › jfb-3984578-supplementary.pdf]

Table S1 sumarisation of Etiology defect, anatomical site of defect, second stage of autografting timing differences between ADM, graft tickness and histological examination

	IDL	ISL	NEVELIA
**1. Etiology of the underlying defect**			
Malign tumors	18	0	5
Benign tumors	6	1	0
Trauma	7	3	5
Ischemic wounds	2	1	2
Acute burns	5	9	3
Postcombustional scars	2	6	0
**2. Anatomical site of biopsy**			
Scalp	5	2	0
Head/neck	9	2	3
Trunk	8	4	1
Upper limb	10	1	6
Lower limb	8	6	3
Foot	2	1	2
Others	2	0	0
**3. Timing of STSG**			
2 WEEKS	20	0	10
3 WEEKS or more	25	15	5
**4. Graft thikness**	0,4 mm	0,4 mm	0,4 mm
**5. Histological/IHC markers assessed (H&E, Masson, CD105, D2-40)**	38	20	14

Table S2 Our results in regard of the literature findings

**Analyzed Variable**	**Our Study Results (N = 75)**	**Results from the Published Literature**	**Comparative Observations / Gap Analysis**
**Median age**	61–65 years, similar across matrices	58–69 years in large ADM cohorts (Integra, Nevelia) [4].	Alignment with international demographic profiles.
**Defect etiology**	Mixed: oncologic (33–60%), trauma (15–33%), burns (5–75%)	Although dermal regeneration templates were initially developed for major burn surgery, current clinical series and systematic reviews show that they are used predominantly for coverage of oncologic and traumatic soft-tissue defects, especially in elderly patients. Early experience with Integra suggested safety in burns involving	The high proportion of burns in the ISL group explains LOS differences.

		<20% TBSA, with later trials extending its use to larger burns [32,33].	
**Anatomical location**	Predominantly limbs, scalp, trunk	Across contemporary dermal regeneration template and ADM literature, the scalp and extremities represent the most frequently reported anatomical sites, driven by oncologic defects in the scalp and traumatic or post-oncologic soft-tissue loss in the limbs [32,34].	Comparable distribution, with a larger "Other/NA" fraction.
**Length of hospital stay (LOS)**	IDL: 3 days; ISL: 18 days; Nevelia: 3 days. Significant unadjusted difference.	Length of stay with dermal regeneration templates is highly etiology-dependent: in life-threatening burns, LOS is typically measured in weeks to months, whereas in selected oncologic or traumatic reconstructions without systemic compromise, LOS can be reduced to a few days [7,35].	High ISL LOS aligns with burn series; after adjustment, matrix type does not independently influence LOS.
**STSG at 14 days (%) vs STSG at ≥21 days (%)**	40% (all matrices) vs 60% (all matrices)	Split-thickness skin grafting after placement of a dermal regeneration template is typically performed after approximately 2–3 weeks, once the neodermis has adequately vascularized and the superficial layer can be safely removed [13,36,37].	Our results are at the upper end, indicating fast integration.
**Healing without STSG**	4% (3 patients)	Some case series and cohort studies note that, in selected patients (particularly those with minor, well-vascularized defects or significant comorbidities warranting avoidance of a second procedure) dermal regeneration templates may occasionally be left to heal by secondary intention without a skin graft, although no aggregated, generalizable, or validated percentage is established in the current literature [38}.	Alignment with the literature, confirming proper case selection.
**Overall complications**	8% (no significant differences between matrices)	10% in ADM cohorts (infection, matrix loss, hypergranulation) [38].	Lower-than-average complication rate, confirming good

			management and patient selection.
**Integration at 14 days**	Most matrices exhibited good integration	Integra and Nevelia studies report full integration at 21-28 days, with neovascularization starting at 1-2 weeks [12, 13].	Relatively matches the timing reported in studies and confirmed histologically.
**Histological integration (H&E, MT, CD105, D2-40)**	Intense vascularization, type I collagen fibers, minimal inflammatory infiltrate, robust neoangiogenesis	Literature describes identical phases at 2–4 weeks: residual porosity, intense vascularization, collagen deposition [12].	Full confirmation of standard ADM integration stages, with more detailed documentation.
